# CoreSlicer: a web toolkit for analytic morphomics

**DOI:** 10.1186/s12880-019-0316-6

**Published:** 2019-02-11

**Authors:** Louis Mullie, Jonathan Afilalo

**Affiliations:** 1 Department of Medicine, McGill University, 3755 Cote Ste Catherine Rd, E-222, Montreal, QC H3T 1E2 Canada; 2 Division of Cardiology, McGill University, Montreal, QC Canada; 3 Centre for Clinical Epidemiology, Lady Davis Institute, Jewish General Hospital, McGill University, Montreal, QC Canada

**Keywords:** Analytic morphomics, Morphometric analysis, Body composition analysis, Planimetric measurements, Medical image segmentation, Computed tomography, Obesity, Sarcopenia

## Abstract

**Background:**

Analytic morphomics, or more simply, “morphomics,” refers to the measurement of specific biomarkers of body composition from medical imaging, most commonly computed tomography (CT) images. An emerging body of literature supports the use of morphomic markers measured on single-slice CT images for risk prediction in a range of clinical populations. However, uptake by healthcare providers been limited due to the lack of clinician-friendly software to facilitate measurements. The objectives of this study were to describe the interface and functionality of CoreSlicer- a free and open-source web-based interface aiming to facilitate measurement of analytic morphomics by clinicians - and to validate muscle and fat measurements performed in CoreSlicer against reference software.

**Results:**

Measurements of muscle and fat obtained in CoreSlicer show high agreement with established reference software. CoreSlicer features a full set of DICOM viewing tools and extensible plugin interface to facilitate rapid prototyping and validation of new morphomic markers by researchers. We present published studies illustrating the use of CoreSlicer by clinicians with no prior knowledge of medical image segmentation techniques and no formal training in radiology, where CoreSlicer was successfully used to predict operative risk in three distinct populations of cardiovascular patients.

**Conclusions:**

CoreSlicer enables extraction of morphomic markers from CT images by non-technically skilled clinicians. Measurements were reproducible and accurate in relation to reference software.

**Electronic supplementary material:**

The online version of this article (10.1186/s12880-019-0316-6) contains supplementary material, which is available to authorized users.

## Background

Syndromes characterized by pathological alterations of body composition, such as sarcopenia, cachexia and obesity, are increasingly prevalent and portend an increased risk of adverse health outcomes. Accordingly, there is a growing interest, both at the clinical and academic levels, in using body composition analysis to identify vulnerable patients who would benefit from targeted evaluation and treatment [1]. Analytic morphomics, or more simply, “morphomics,” refers to the measurement of specific biomarkers of body composition from medical imaging, most commonly computed tomography (CT) images [2–4]. An emerging body of literature supports the use of morphomic markers measured on single-slice CT images for risk prediction in a range of clinical populations (Table 1). The analytic morphomics group at the University of Michigan has had a pioneering influence in the field and has undertaken a major standardization effort with the publication of reference values derived from a large population of patients [5]. Despite promising results, adoption by healthcare providers has been limited due to the lack of clinician-friendly software to facilitate measurements.

**Table 1:** Selected single-slice morphomic markers derived from muscle and adipose tissue area measurements on thoracic and abdominal CT scans

Category	Marker name and definition	Segmentation algorithms	ID
Muscle *(lumbar)* − 29 to + 150 HU [24]	Psoas muscle area: combined area of the right and left psoas muscles, in mm^2^ [25–47].	Shape model [48, 49]	PMA
	Psoas muscle attenuation: mean attenuation value within the psoas muscles, in HU [31, 46, 50–55].	–	PMA_HU
	Lumbar dorsal muscle area: combined area muscle contained within the region posterior to the spine and ribs, no more lateral than the lateral-most edges of the erector spinae muscles (includes latissimus dorsi, quadratus lumborum, and erector spinae muscles), in mm^2^ [56–59].	Atlas-based [60], thresholding-based [61], fuzzy C-means [62, 63]	LDMA
	Lumbar dorsal muscle attenuation: mean attenuation value within the dorsal muscles, in HU [63, 64].	–	LDMA_HU
	Total lumbar muscle area: combined area of the psoas, rectus abdominis, pyramidalis, transversus abdominis, internal and external oblique, plus the dorsal muscle area, in mm^2^ [31, 56, 64–66]	FEM-based [67, 68]	TLMA
	Total lumbar muscle attenuation: mean attenuation value within the lumbar muscles, in HU [69].	–	TLMA_HU
Muscle *(thoracic)* − 129 to + 150 HU	Total thoracic muscle area: combined area of the pectoralis, intercostal and paraspinal muscles, in mm^2^ [70–73].	FEM-based [74]	TTLMA
	Total thoracic muscle attenuation: mean attenuation value within the lumbar muscles, in HU (ND).	–	TTLMA_HU
Fat *(lumbar)* −190 to − 30 HU	Visceral fat area: total area of intraperitoneal fat, in mm^2^ [31, 54, 55, 75–79].	Fuzzy C-means [80], fuzzy affinity [81], thresholding [82], separation mask [83], polar projection ([84], edge linking [85], other [86]	VFA
	Visceral fat attenuation: mean attenuation value within the visceral fat, in HU [51, 53, 87–93].	–	VFA_HU
	Subcutaneous fat area: total area of fat tissue between the skin and abdominal/back wall, in mm^2^ [31, 51, 54, 55].	FEM-based [68], separation mask [93], other [93]	SFA
	Subcutaneous fat attenuation: mean attenuation value within the subcutaneous fat, in HU [53, 87–91].	–	SFA_HU
	Total abdominal fat area: combined area of visceral and subcutaneous fat tissue, plus intramuscular fat, in mm^2^ [93].	–	TAA
	Total abdominal fat attenuation: mean attenuation value within the abdominal fat, in HU (ND).	–	TAA_HU
Fat (thoracic) − 190 to − 30 HU	Epicardial fat area: fat located between the heart and the pericardium, in mm^2^ [94–100].	Random forest [101, 102], geodesic active contours [103], fuzzy C-means [104], other [105]	EFA
	Epicardial fat attenuation: mean attenuation value within the epicardial fat, in HU [106]	–	EFA_HU

**Fig. 1:**
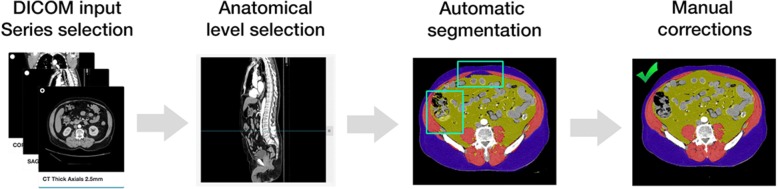
Typical workflow for measurement of analytic morphomics

Figure 1 displays a typical workflow for determination of morphomic markers; which begins by opening a study in DICOM format, selecting a validated reference anatomical level on a reconstructed view (e.g. the level of the 4th lumbar vertebra on a sagittal view), visualizing the corresponding axial image, and finally performing measurements using a combination of automatic and manual segmentation tools. Markers that can be extracted using this workflow include tissue areas (muscle, fat, solid organs, bone), as well as attenuation values in Hounsfield units (HU), which provide an index of tissue composition or “quality.” Key advantages over other methods of body composition assessment include the wide availability of existing CT datasets, the ability to perform measurements retrospectively, and the ability to accurately assess tissue quantity, quality and distribution with high reproducibility and minimal assumptions.

Table 1 displays selected examples of morphomic markers of muscle and adipose tissue derived from single-slice measurements on abdominal or thoracic CT scans, with accompanying references to clinical validation studies. The wide range of applications illustrates the versatility of the proposed workflow. Automated segmentation algorithms are referenced when available.

Given the increasing availability of clinically indicated CT imaging studies [6] and the mounting recognition of morphomics as a prognostically relevant method of body composition assessment, it is highly desirable to facilitate measurements for researchers and clinicians. However, developing software for the morphomics community poses unique development challenges because the field exists at the confluence of multiple scientific disciplines, including medical image analysis (MIA), body composition research, clinical medicine, and epidemiology. We have identified four key challenges and corresponding design requirements for software to bring morphomics “to the bedside” and to a larger community of researchers (Table 2). In this section, we review existing MIA software in light of these design constraints and identify pertinent limitations of established approaches.

**Table 2:** Design objectives for translational morphomics software

Design objective	Rationale
Clinician-friendly, goal-directed interface	Clinicians may not have the time and technical know-how required to use professional medical image analysis software.
Cross-platform support, minimal or no install required	Researchers and clinicians collaborating on morphomics projects across institutions are likely to work in different computer system environments. Clinicians may be performing measurements on work machines where MIA software has not been installed.
Extensibility via cloud-enabled plugins	A flexible plugin interface enables application of the software to a wider variety of use cases, and cloud abilities facilitate the processing of large datasets.
Free license and open source codebase	An open-source codebase and reuse-friendly license contributes to project sustainability by allowing contributions from other researchers.

A first key challenge is to bridge the gap in technical knowledge between body composition researchers, who develop and validate morphomic markers, and healthcare providers who can utilize these markers to enhance their evaluation and treatment of patients. The ability quickly review and edit results is crucial in the clinical setting, where expert validation of computer-generated data is considered standard of care. Thus, for morphomics data to translate into improved decision-making, a streamlined, “clinician-friendly” interface for reviewing results is essential.

Current MIA software packages have adopted the “toolbox” approach of user interface organization. This approach is flexible, yet results in a high level of interface complexity due to the multiplicity of controls and menus. For example, the main user control interface includes 27 visible clickable control elements in Nora [7], 28 in ImageJ [8], and 32 in slice-O-Matic [9]. By contrast, the “wizard” approach is an organizational pattern that presents essential controls in step-by-step fashion, which facilitates the execution of unfamiliar tasks. The wizard or “workflow-oriented” approach is most useful for “a non-expert user [who] needs to perform an infrequent complex task consisting of several subtasks, where decisions need to be made in each subtask.” [10] While the workflow-oriented pattern may be a valuable approach for clinician-facing MIA software, only one of the major packages evaluated has adopted this approach as a primary user interface organization pattern, and this software did not support workflows for measurement of analytic morphomics [11].

A second key challenge is cross-platform compatibility, which is crucial to address owing to the heterogeneity in computing environments deployed by teams of researchers and clinicians working across different institutions. Of the major currently existing tools, Slice-O-matic, Materialize [12] and Segment [13] only offer Windows support, while Osirix only supports Mac environments [14]. 3DSlicer [15], ImageJ [15] and ITKSnap [16] are notable free options with good cross-platform compatibility. Although extremely powerful, their interfaces have not been adapted to facilitate measurements by non-clinicians with little technical knowledge in image analysis.

Most current MIA software packages (e.g. ImageJ, Segment, Osirix, 3DSlicer) are distributed via desktop apps that are installed on end-user machines, and these require continuous updating to ensure forward-compatibility. Yet, clinicians may be performing measurements on work machines where MIA software has not been installed, and, in many cases, institutional restrictions prevent end-users from installing such software in the clinical environment. Over the last decade, browser applications, or “web apps,” have emerged as a more sustainable means of achieving robust cross-platform independence; these can be used instantly on any machine connected to the Internet, without prior installation. Nora is an example of an MIA software with an emphasis on brain imaging that is written entirely as a browser application [7]. Nora provides many advanced analysis features; limitations of this project are the lack of a plugin architecture, moderate to high interface complexity, and lack of a formal open-source development process.

A third challenge is to facilitate prototyping, validation and adoption of algorithms to segment scans and measure morphomic markers. Notably, at the time of writing, none of the segmentation algorithms presented in Table 1 had been released as a plugin compatible with freely available MIA software. While some MIA software have support for language-specific plugins (e.g. Java for ImageJ, Objective C for Osirix, Matlab for Segment, Python and C++ for 3DSlicer), the lack of cross-talk between these languages hinders the development of a robust plugin ecosystem. A modern method of providing extensibility via “plugins” is the use of HTTP application programming interfaces (APIs), which enable the developer to write plugins in any language capable of running a simple web server. As an added benefit, computational resources in the cloud can be leveraged with the same interface and ease of use as local plugins. The neuroimaging community has already adopted cloud-based tools such as MRIcloud [17], CBRAIN [18] and VolBrain [19] to facilitate collaboration and sharing of computational resources across geographically dispersed research groups. Given the large-scale nature of the data sets used in morphomics, the community would benefit from a platform that enables rapid prototyping and deployment of morphomics algorithms in the cloud.

Finally, a free licensing model and an open-source development process was chosen as a design goal to facilitate greater adoption of the software amongst researchers, and to promote project sustainability by allowing developers to contribute their own updates and plugins. Given that the adoption of commercial MIA software imposes a financial limit on wider-scale collaboration, it is in the multidisciplinary spirit of the morphomics community to involve all stakeholders in building free, open-source alternatives that are equitably available to a global audience of clinicians irrespective of cost.

As summarized in Table 3, a review of existing MIA software identified that none satisfactorily addressed all of the key design constraints that we have proposed to guide development of translational morphomics software. While many are comprehensive and powerful tools, none of the currently available software tools provides a simple, clinician-friendly pipeline to facilitate morphomic analysis. To address this unmet need, we introduce the first web-based interface optimized for measurement of morphomics, called CoreSlicer, which is publicly available free of charge at https://www.coreslicer.com. Users can extend the software with their own plugins running on their own machine or on cloud services through HTTP endpoints. The source code to the interface is available on GitHub, and allows users to run the application on their local machines. In this paper, we discuss the structure and functionality of CoreSlicer, validate its results against reference software, and discuss published studies illustrating its relevance for clinicians and researchers.

**Table 3:** Selected major medical image analysis tools potentially suitable for morphomic analysis, features and limitations

Project name and URL	Workflow-oriented	Web interface	Platform independent	Plugin interface	Web plugins	Free license	Open source
Slice-o-matic http://www.tomovision.com/products/sliceomatic.html	N	N	N (Windows-only)	N	N	N	N
ImageJ https://imagej.nih.gov/ij/	N	N	Y (Java app)	Java only	N	Y	Y
Materialize http://www.materialise.com/en/medical/software/mimics	N	N	N(Windows-only)	N	N	N	N
Segment https://github.com/Cardiac-MR-Group-Lund/segment-open/	N	N	N (Windows-only)	Matlab only	N	Y	Y
MIA http://mia.sourceforge.net/	Y	N	N (POSIX-only)	C++ only	N	Y	Y
ITKSnap http://www.itksnap.org/pmwiki/pmwiki.php	N	N	Y (binaries)	C++ only	N	Y	Y
3DSlicer https://www.slicer.org/	N	N	Y (binaries)	Python C++	N	Y	Y
Osirix http://www.osirix-viewer.com/	N	N	N (Mac only)	Objective C only	N	Y	Y
Nora http://www.nora-imaging.com/	N	Y	Y	N	N	Y	N
CoreSlicer https://www.coreslicer.com	Y	Y	Y	Any language	Y	Y	Y

### Implementation

#### Specifications of CoreSlicer

CoreSlicer is a browser-based application and is written entirely in Javascript, conformant to the ECMAScript 5 specification. A public running instance of CoreSlicer is provided free of use at www.coreslicer.com. This public running instance showcases elemental plugins for segmentation of abdominal structures (Additional files 1 and 2). Users may also download a standalone version of CoreSlicer free of charge at https://github.com/louismullie/coreslicer. The standalone version of CoreSlicer is released under the MIT license.

#### Testing requirements

CoreSlicer is developed for and was tested on Google Chrome, versions 65.0 and above. The Google Chrome platform was selected due to its widespread adoption and stability across multiple operating systems. Other browsers are not currently supported. Tests were conducted on a machine with a 2 GHz intel Core i5 processor and 8 GB of rapid-access memory (RAM). A minimum 1 GB of RAM is recommended to load full-body CT scans at 2.5 mm slice thickness, or 2 GB RAM at 1 mm slice thickness. A minimum processor speed of 1.0 GHz is recommended to support interface drawing functions.

#### Program structure

**Fig. 2:**
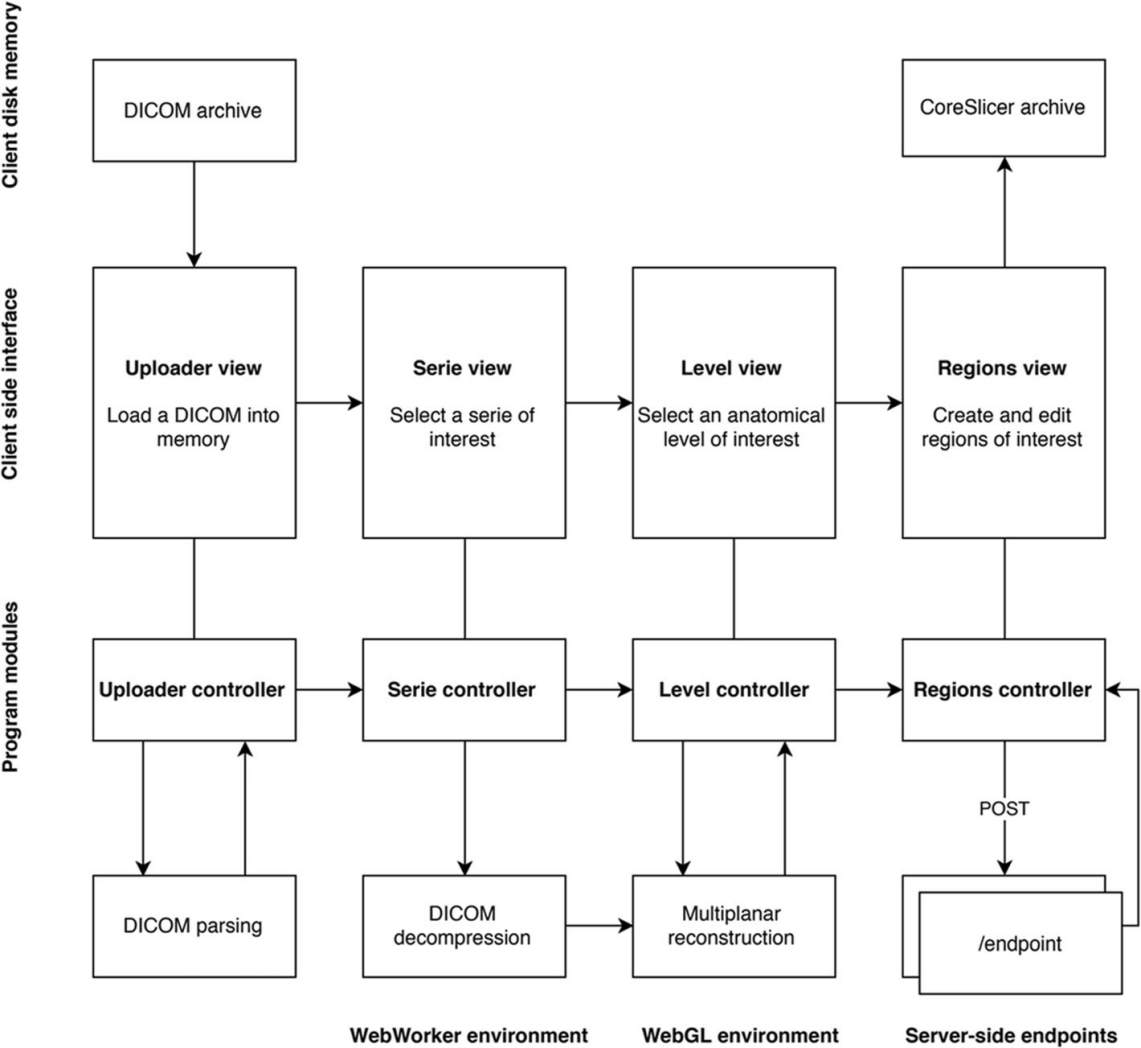
Program structure overview

CoreSlicer is divided into 4 main modules (Fig. 2). As is common for web applications, a “model-view-controller” architecture is employed to enforce separation of concerns between different application modules [20]. The “Uploader” controlled provides functionality for loading DICOM files from disk. The “Series” controlled handles selection of a series of interest within a DICOM file and decompression of the DICOM file, if necessary. The “Level” controlled provides functionality to select a level of interest on a sagittal reconstruction. The “Regions” controller provides functionality for displaying DICOM images and creating and editing regions of interest (ROIs).

#### Graphical user interface

**Fig. 3:**
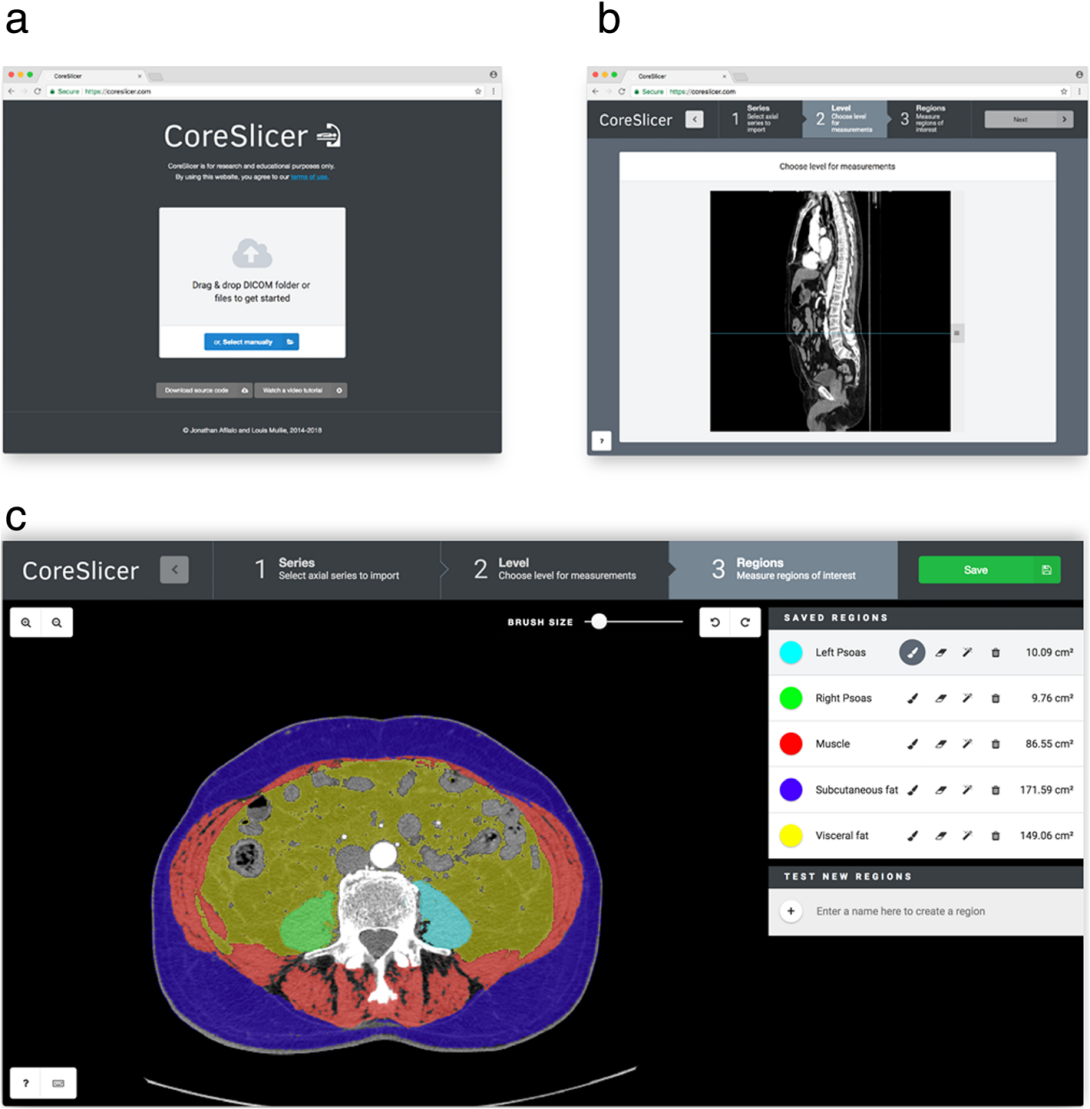
Graphical user interface overview. Panel **a** shows the “Uploader” window, where DICOM archives can be imported. Panel **b** shows the “Level” window, using which an anatomical level can be selected. Panel **c** shows the “Region” window, using which regions of interest can be segmented

The CoreSlicer interface is organized into 4 main windows (Fig. 3). From the “Uploader” window (Fig. 3a), users can load one or more DICOM file(s) by dragging and dropping or via the operating system’s native file select input. From the “Series” window, users can select a series of interest among those contained in the DICOM archive. From the “Level” window (Fig. 3b), users can select a level of interest on a sagittal reconstruction image of the previously selected series. From the “Region” window (Fig. 3c), users can define one or more ROIs, and assign a custom HTTP endpoint that provides automatic segmentation functionality for the relevant ROI. Users can draw and edit an ROI using the threshold brush and eraser tools. Results are exported to a ZIP archive containing the segmentation masks as well as area and Hounsfield unit measurements in comma-separated value (CSV) format.

#### Extensibility via plugins

**Fig. 4:**
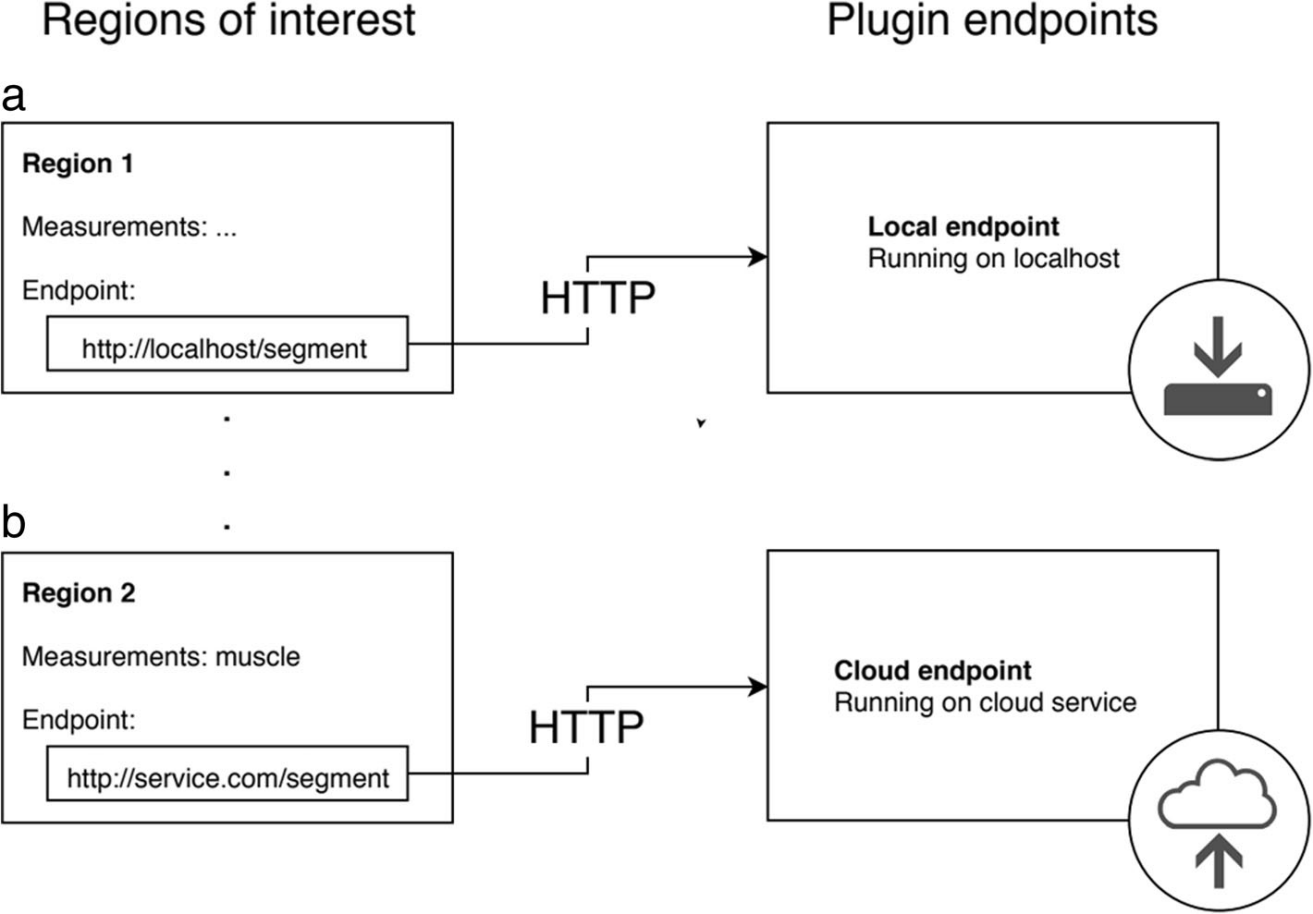
Plugin architecture overview. Panel **a** shows an example of a plugin served on a local endpoint. Panel **b** shows an example of a plugin served on a remote endpoint

CoreSlicer can be extended via user-supplied webhooks that provide segmentation functionality over an HTTP interface. CoreSlicer’s plugin API structure is illustrated in Fig. 4.

From the “Regions” window, when creating a new custom region, CoreSlicer allows users to assign a custom HTTP webhook that is triggered when a user initiates the draw action assigned to the corresponding ROI. In response, a POST request is sent to the assigned endpoint containing the anonymized DICOM image binary data and additional slice information encoded as a multipart FormData object (request MIME type multipart/form-data). User endpoints return a binary segmentation mask in PNG format (response MIME type image/png), where all non-zero pixels are assigned a value of 1. Further information is available from the user manual, which can be found on Github (https://github.com/louismullie/coreslicer).

#### Local deployment

Deployment on a local server can be performed with minimal technical knowledge using Node.js, an open source programming runtime that is supported by all major operating systems including Windows, Mac OS and UNIX. Local deployments can be updated to the latest public version of CoreSlicer’s code via a single command, using the Git version management system. A detailed guide on how to install a local deployment and retrieve updates is available at https://github.com/louismullie/coreslicer.

#### Implementation challenges

Although highly flexible and granular, the DICOM standard is complex to manipulate because it supports multiple numerical representation, data sorting and compression formats. Cornerstone.js, an open-source DICOM utility library, was used to parse DICOM attributes and render DICOM images. A custom algorithm was implemented to reorder slices based on their position in space as determined from DICOM attributes. Jpx.js, an open-source JPX decompression implementation forked from Mozilla’s PDF.js project, was used to provide support for decompression of DICOM archives.

While browser-based applications are highly advantageous for cross-platform compatibility, they represent a “resource-costly” environment in which large file manipulation are memory-expensive operations. The embarrassingly parallel nature of DICOM slice decompression enabled the use of concurrency to increase processing speed by distributing data to a pool of 8 WebWorker processes running simultaneously. To further optimize performance, CoreSlicer adheres to the “lazy loading” design pattern, deferring the initialization of memory-costly data structures until the point at which the data is needed for user interface rendering.

In addition to performance limitations with regards to manipulation of large files, browser environments have limited native abilities for complex numerical operations. To provide fast multiplanar reconstruction (MPR) for CoreSlicer, we implemented an MPR algorithm using WebGL, an HTML5 standard that allows for accelerated manipulation of 3-dimensional data on the graphics processing unit (GPU). To store DICOM images in memory and manipulate them, CoreSlicer leverages TypedArray objects, which provide a fast mechanism for accessing raw binary data in Javascript, and which can be easily transferred to the GPU using WebGL. The combination of these strategies allows CoreSlicer to efficiently reslice full-body CT scans, with minimal computational time, on the order of < 500 ms.

To enable smooth drawing operations for manual labeling of DICOM images, CoreSlicer leverages Canvas API, which enables hardware-accelerated rendering of two-dimensional graphics in the browser. A system of stacked Canvas objects is used to represent multiple overlying layers in transparency. Tegaki.js, an open-source HTML drawing library, is employed as a foundation for CoreSlicer’s customized DICOM drawing tools.

#### Security and privacy

Ensuring the security and privacy of user data is a key implementation challenge in any web environment. The core application functionality of CoreSlicer is implemented entirely on the client machine and does not require any transmission of data. This includes loading DICOM files, decompressing image files if needed, performing multiplanar reconstruction, as well as manually drawing and editing measurements. The use of optional segmentation plugins requires transmission of information over the Internet. This information is protected in transit using RSA encryption with 4096-bit keys, via the Secure Sockets Layer (SSL) protocol. In addition, through its plugin interface, CoreSlicer allows users to securely transmit data directly from their machine to user-controlled processing endpoints, without any information transiting via CoreSlicer’s servers. Users who desire enhanced privacy can run the CoreSlicer interface on their local machines. CoreSlicer automatically strips DICOM files of identifying information prior to any form of external transmission in order to prevent disclosure of protected health information.

#### Validation methods

Measurements from CoreSlicer were validated against slice-O-matic, a commercial cadaver-validated reference software. Cross-sectional area measurements (Fig. 4) were performed by two trained observers (LM and JA), respectively designated observer A and observer B. Observer A performed measurements of VFA, SFA, TLMA and PMA in triplicate, once using the slice-O-matic software package (version 5.0), and twice using the manual drawing tools in the CoreSlicer web interface (version 1.0). Repeated measurements were performed at least 1 week apart to limit observer bias. In a subset of 20 scans, observers A and B each obtained measurements of VFA, SFA, TLMA and PMA using CoreSlicer. All measurements were performed on axial series at the superior aspect of L4, immediately below the vertebral endplate.

**Fig. 5:**
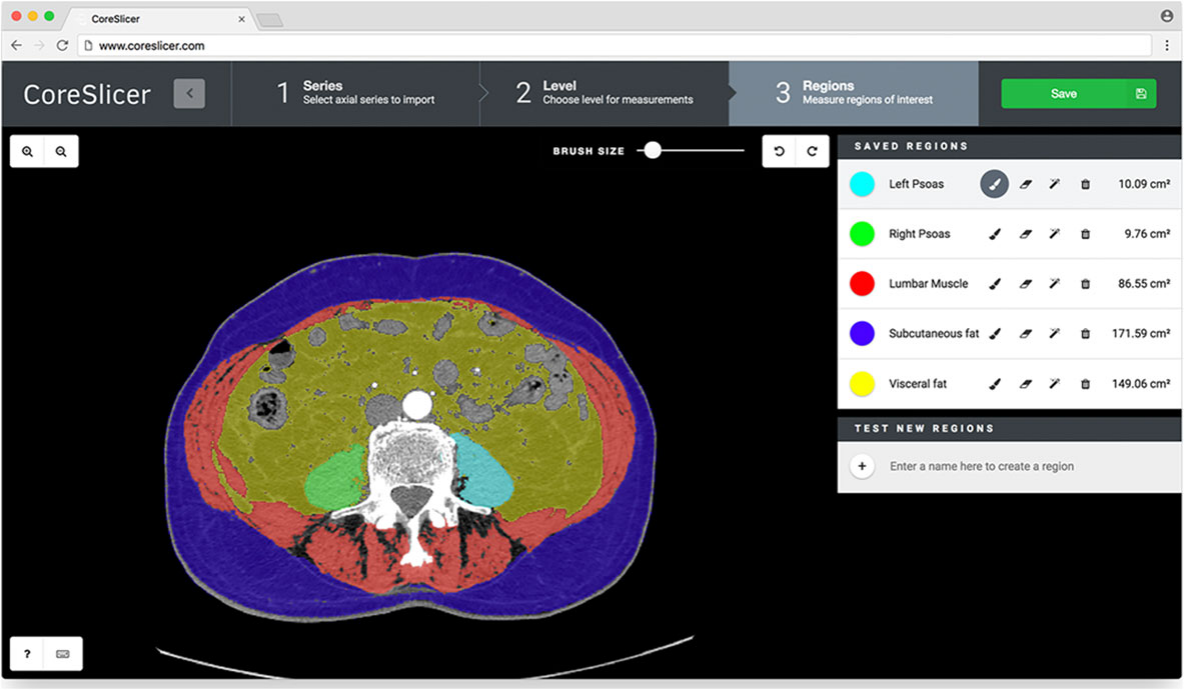
Illustration of muscle and fat segmentation at L4 in CoreSlicer

ROIs were defined as: psoas muscle area (PMA), total lumbar muscle area (TLMA), visceral fat area (VFA) and subcutaneous fat area (SFA). These were chosen among markers illustrated in Table 1 based on the robustness of the supporting clinical evidence. A representative labeled image is illustrated in Fig. 5. Hounsfield unit ranges were − 190 to − 30 for adipose tissue (VFA and SFA), and − 29 to 150 for skeletal muscle (TLMA and PMA) [9].

#### Statistical analyses

For comparisons between sets of measurements, mean difference, 95% level of agreement (LOA), and Spearman’s correlation coefficient were calculated. Results are presented using Bland-Altman plots (Additional file 3). Additionally, for the main outcome of interest, which compared measurements in CoreSlicer with measurements in slice-O-matic, the intraclass correlation coefficient was calculated, and power analysis was performed. Assuming a hypothesized ICC of 0.99, and a null ICC value of 0.90, a sample of 50 subjects (2 measurements per subject) has 100% power to detect a difference between the two measurement methods at a 5% type 1 error rate. All statistical analyses were performed with the STATA software package (version 14.0, College Station, Texas).

## Results

A total of 50 CT scans were drawn and analyzed from a cohort of older adults undergoing a routine pre-operative CT scans during assessment for a heart valve procedure. The population comprised 24 women and 26 men, with a mean age of 80 years (range 64 to 96 years). The mean BMI was 26.2 (range 20.5 to 44.5). Descriptive statistics of the study population are presented in Table 4.

**Table 4:** Descriptive statistics of the study population

Variable	Males (*N* = 26)	Females (*N* = 24)
Age (y)	81.4 ± 7.6 (64–96)	79.8 ± 7 (67–92)
Height (m)	1.7 ± 0.1 (1.6–1.9)	1.6 ± 0.1 (1.5–1.8)
Weight (kg)	75.7 ± 10.4 (54.5–100)	67.4 ± 15.3 (48.0–99.0)
BMI (kg / m^2^)	26.1 ± 2.8 (20.2–34.2)	26.2 ± 5.8 (21.5–44.5)
VFA (cm^2^)	252.5 ± 122.7 (114–603.9)	252.5 ± 122.7 (114–603.9)
SFA (cm^2^)	198.9 ± 62.9 (113.3–368.3)	208.6 ± 101.9 (89.4–584.6)
TLMA (cm^2^)	134.5 ± 22.3 (87.1–173.4)	102.7 ± 20.8 (80.0–155.7)

**Fig. 6:**
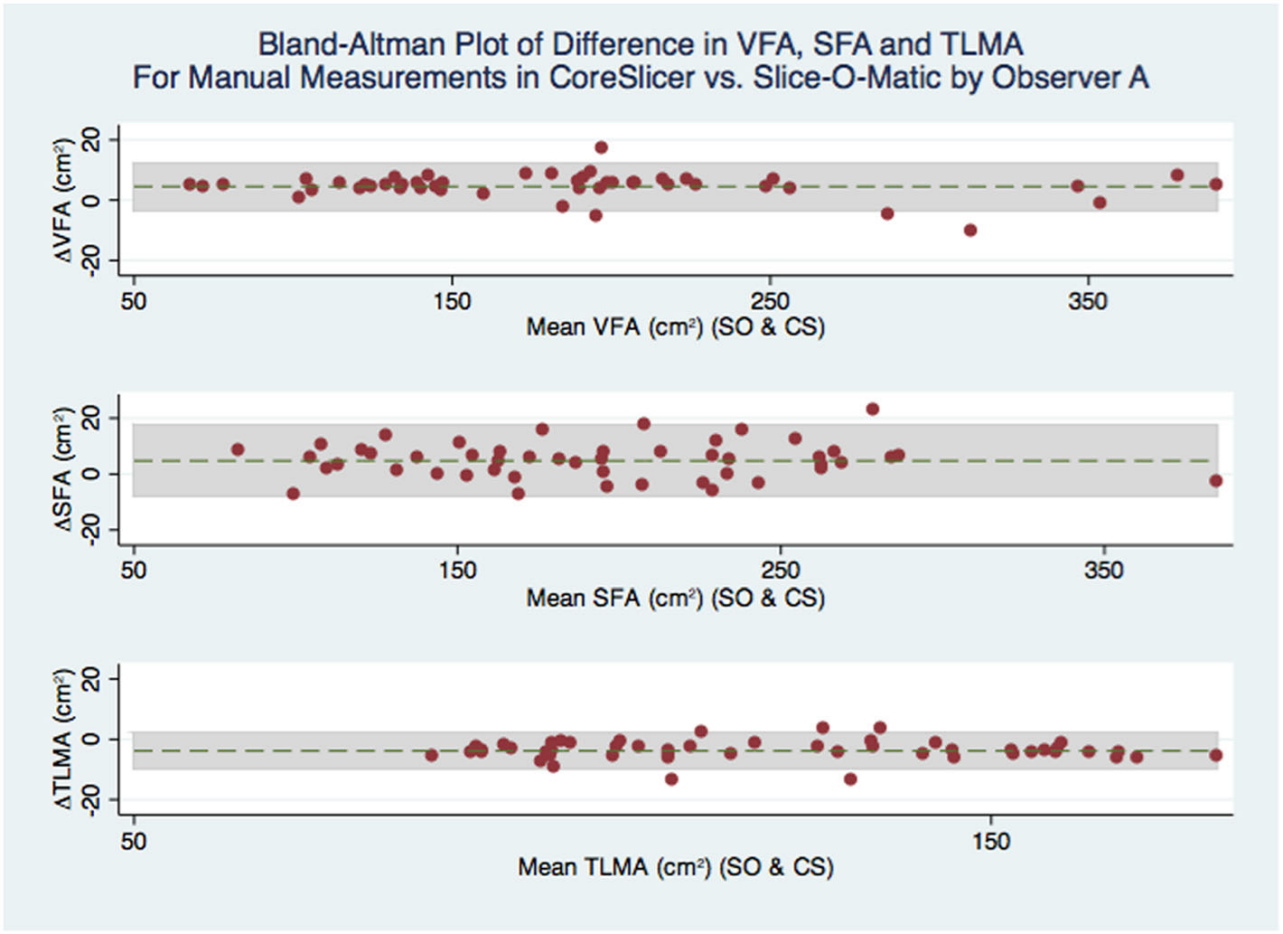
Bland-Altman plot of difference in VFA, SFA and TLMA for manual measurements in CoreSlicer vs. Slice-O-Matic by Observer A

Mean cross-sectional areas were 202.6 ± 82.5 cm^2^ for VFA, 214.2 ± 105.8 cm^2^ for SFA, 119.05 ± 26.5 cm^2^ for TLMA, and 19.77 ± 5.6 cm^2^ for PMA. In Fig. 6, a Bland-Altman analysis is presented for comparison of cross-sectional area measurement differences between CoreSlicer and Slice-O-Matic. Mean absolute differences in cross-sectional areas were 4.2 cm^2^ (− 4.7 to 13.1 cm^2^) for VFA, 4.8 cm^2^ (− 7.7 to 14.7 cm^2^) for SFA, − 3.8 cm^2^ (− 10.1 to − 2.4 cm^2^) for TLMA, and 0.4 cm^2^ (− 1.4 to 2.2 cm^2^) for PMA. Mean differences in cross-sectional areas expressed as relative percentages were 2.1% (− 2.4 to 6.5%) for VFA, 2.3% (− 3.9 to 7.3%) for SFA, − 3.0% (− 7.9 to 1.9%) for TLMA and 2.0% (− 7.0 to 11.0%) for PMA. Intra-class correlation coefficients were 0.998 for VFA, 0.999 for SFA, 0.984 for TMA, 0.994 for PMA. Spearman’s correlation coefficient exceeded 0.99 for all ROIs, with *p* values of < 0.001.

**Fig. 7:**
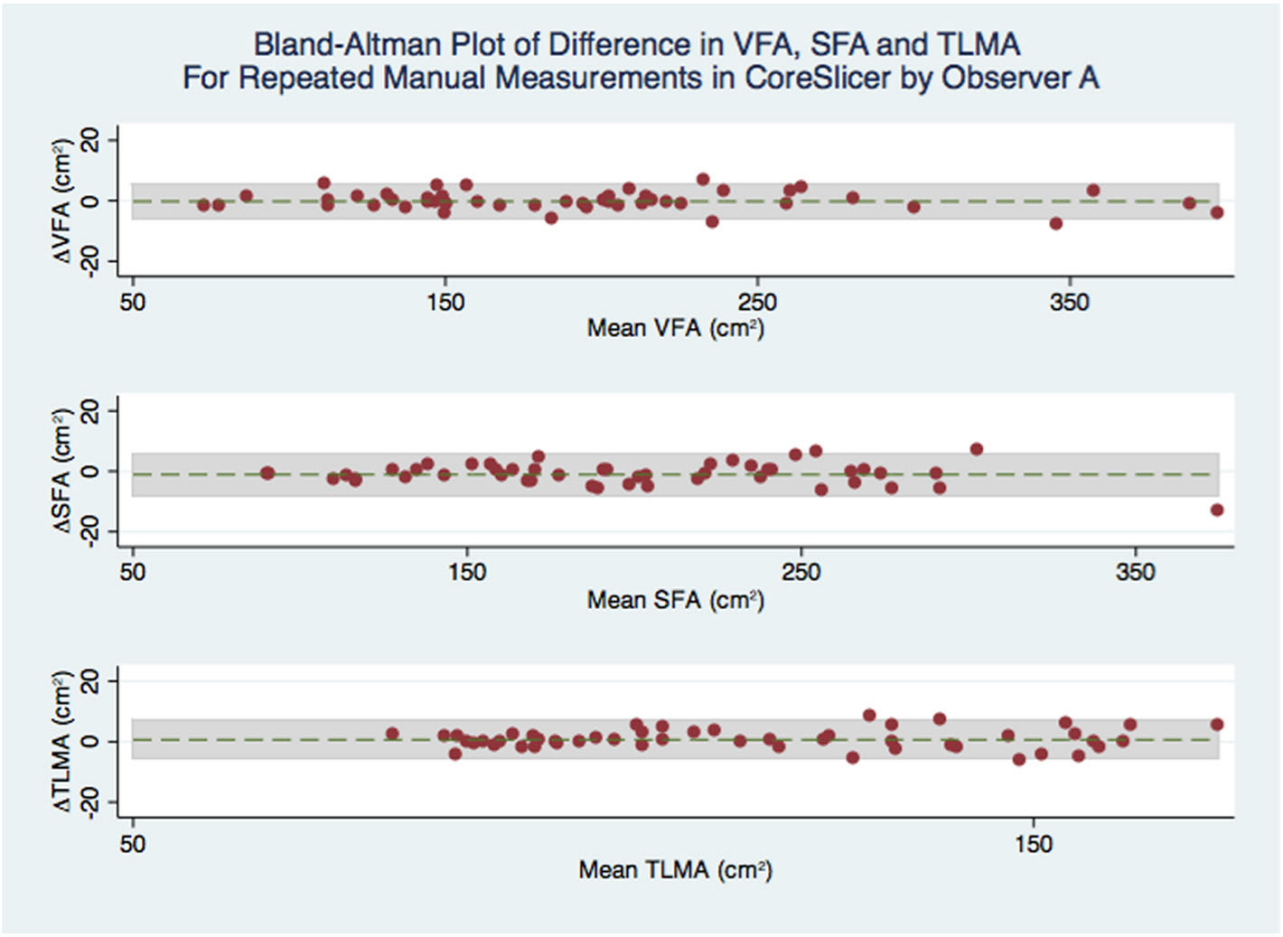
Bland-Altman plot of difference in VFA, SFA and TLMA for repeated manual measurements in CoreSlicer by Observer A

**Fig. 8:**
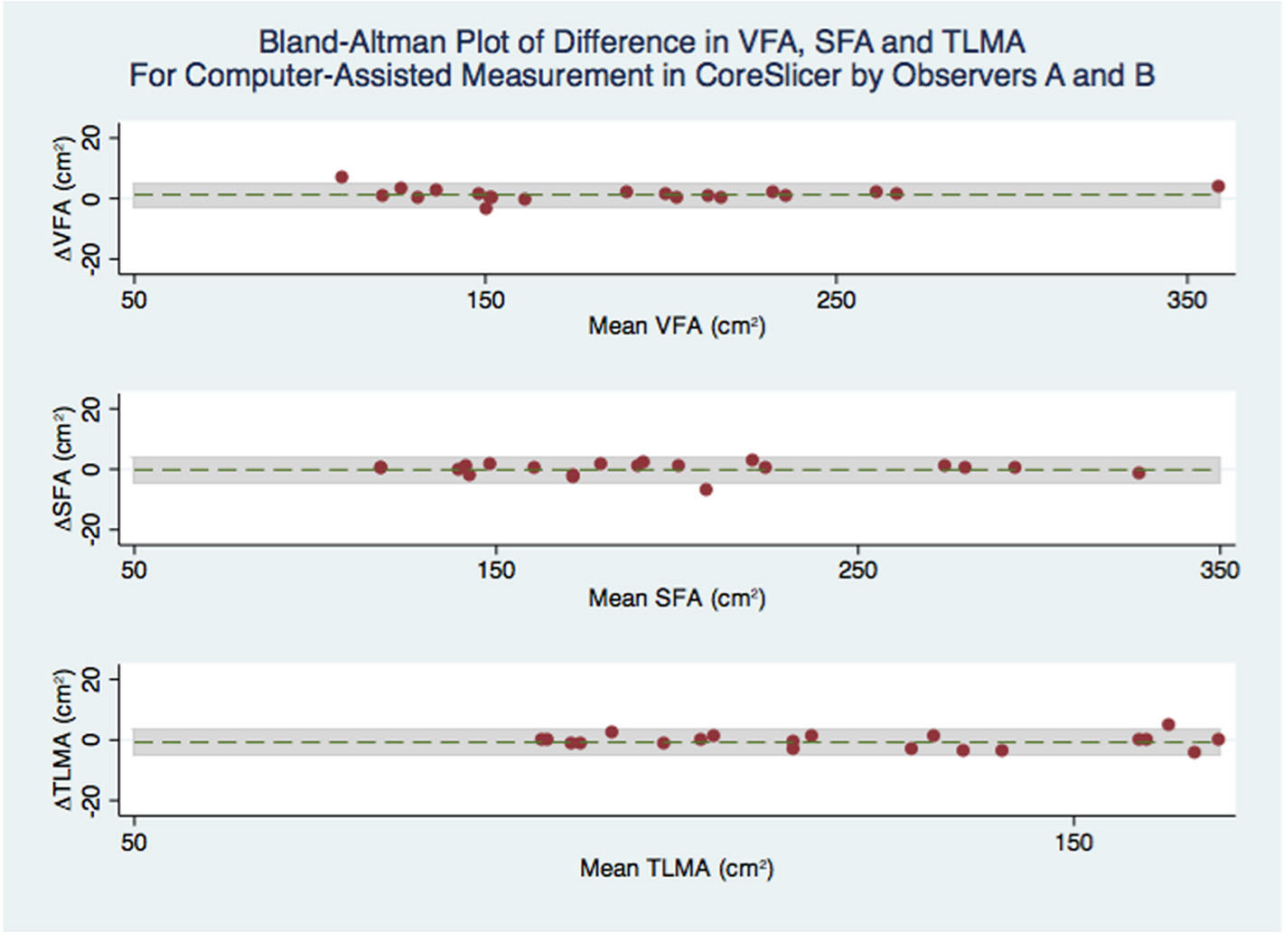
Bland-Altman plot of difference in VFA, SFA and TLMA for computed-assisted measurements in CoreSlicer by Observers A and B

In Figs. 7 and 8, Bland-Altman analyses are shown for intra- and inter-observer measurements in CoreSlicer. For repeated manual measurements by observer A, mean absolute differences in cross-sectional areas were − 0.2 cm^2^ (− 6.0 to 5.8 cm^2^) for VFA, − 1.1 cm^2^ (− 8.0 to 5.8 cm^2^) for SFA, 0.4 cm^2^ (− 1.7 to 2.4 cm^2^) for TLMA, and 0.6 cm^2^ (− 5.7 to 7.0 cm^2^) for PMA. For comparison of automated measurements plus manual corrections between observers A and B, mean differences in cross-sectional areas were 1.2 cm^2^ (− 2.7 to 5.6 cm^2^) for VFA, − 0.2 cm^2^ (− 4.4 to 4.0 cm^2^ for SFA), − 0.8 cm^2^ (− 5.2 to 3.7 cm^2^) for TLMA, and − 0.5 cm^2^ (− 0.9 to 1.8 cm^2^) for PMA.

## Discussion

In this study, we introduced the CoreSlicer web interface and software toolkit for analytic morphomics on CT scan images. CoreSlicer is the first open-source web-based MIA designed and optimized specifically for analytic morphomics. Given its implementation as a browser application, CoreSlicer is portable across all major operating systems (Windows, MacOS, Linux and Chrome OS), addressing an important limitation of several existing tools and facilitating collaboration between researchers working on different platforms. A core set of tools required to manually review and correct segmentation results is provided within the interface, and the workflow is organized in a simple to use, step-by-step wizard. The average time required in CoreSlicer for measurement of psoas muscle area, one of the most robustly validated morphomic markers, was 22 ± 3 s, supporting the benefit of a streamlined, workflow-oriented interface.

Implementation challenges were addressed with state of the art browser application technologies including distributed processing of large files using WebWorkers, hardware-accelerated 2D drawing using the Canvas object, 3D multiplanar reconstruction on the GPU using WebGL, and memory optimizations using TypedArrays.

Researchers can use CoreSlicer to rapidly prototype image segmentation tools in the language of their choice using a standardized HTTP interface. Additionally, CoreSlicer is the first web-based MIA software with out-of-the-box support for asynchronous plugin execution, enabling the use of segmentation tools served in the cloud at the click of a button. For researchers seeking to expand, refine, or adapt the capabilities CoreSlicer, the source code has been made available via an open source repository.

We conducted duplicate measurements of well-validated fat and muscle morphomic markers using CoreSlicer and slice-O-matic, and demonstrated good agreement between the two methods. The limits of agreement were comparable with previously reported values for comparison of the NIH ImageJ and Slice-O-Matic software packages [8]. The intra- and inter-observer variability of measurements was also comparable with previously reported results [21], with the relative differences all being < 3% and unlikely to be clinically significant. Thus, measurements of VFA, SFA and TLMA in CoreSlicer were reproducible and accurate in relation to reference software.

The potential impact of CoreSlicer as a translational tool for morphomics has been demonstrated in three studies aiming to validate the prognostic value of PMA in cardiac and vascular surgery patients. Our work and that of others has shown that psoas muscle area (PMA), a surrogate of lean muscle mass and lower extremity strength, correlates with clinical frailty scores and is an important risk factor for morbidity and mortality following invasive procedures (Table 1). Mamane et al. [32] showed that PMA measured using CoreSlicer was predictive of mortality in elderly women undergoing TAVR. Drudi et al. [38] similarly showed that PMA was predictive of mortality in patients undergoing abdominal aortic aneurysm repair. Zuckerman et al. [30] used CoreSlicer to show that PMA correlated with length of stay following major cardiac surgery. Thus, measurements of PMA in CoreSlicer were incrementally predictive of adverse health outcomes in vulnerable patients undergoing invasive procedures. These measurements could be obtained by medical trainees with no prior medical image analysis training in under 1 min.

These examples illustrate how morphomics can empower healthcare professionals with prognostic information to tailor treatment strategies and individualize care. Patients found to have low muscle mass, often unrecognized by the de facto “eyeball assessment”, may benefit from targeted interventions such as exercise therapy and nutritional supplementation to build muscle mass and strength and minimize their risk of failed recovery after an illness or surgery [22]. This information, which has been consciously absent from the clinical arena owing to the inaccessible nature of its measurement tools, is now readily obtainable within < 1 min at the point of care by healthcare professionals with minimal pre-training.

### Limitations

The results of this study must be considered in light of the following limitations. First, since CT incurs ionizing radiation and is not appropriate for the sole purpose of body composition analysis, CoreSlicer is currently limited to the analysis of images that have been acquired for clinical purposes. Protocols are in development to acquire limited slices with lower radiation. Second, although the CoreSlicer software has been designed to visualize DICOM images produced by both CT and MRI scanners, only the former modality was tested and validated in this initial release of the software. Given the widespread availability of clinically indicated CT studies, we believe that this in no way limits the potential large-scale impact of our software. Third, CoreSlicer is currently capable of performing measurements on one slice per study and is therefore not amenable to compute volumetric measurements in its current iteration. Importantly, the aims of the CoreSlicer project are not to provide a comprehensive MIA or image segmentation toolkit, but rather to provide a streamlined workflow for analytic morphomics, and to maintain the simplicity to achieve our translational goal of a clinician-friendly interface. A large number of single- slice morphomic markers have already been validated and can be measured using CoreSlicer. Once careful planning identifies how to optimally integrate multi-slice functionality while keeping the interface complexity at a minimum, three-dimensional visualization will be implemented using existing open-source libraries [23].

## Conclusions

CoreSlicer is a free and open-source web-based interface aiming to facilitate measurement of analytic morphomics on DICOM images by non-technically skilled clinicians. CoreSlicer features a full set of DICOM viewing tools and extensible plugin interface to facilitate rapid prototyping and validation of new morphomic markers by researchers. In this study, the CoreSlicer interface and functionality is described, and validity of CoreSlicer measurements is established by comparing its results with reference software on a set of 50 abdominal CT scans, demonstrating good reproducibility and agreement with reference software. We present published studies illustrating the clinical relevance of morphomic measurements obtained in CoreSlicer in three distinct populations of cardiovascular patients.

### Availability and requirements

**Project name:** CoreSlicer.

**Project home page:** www.coreslicer.com

**Operating system(s):** platform independent.

**Programming language:** Javascript.

**Other requirements:** none.

**License:** MIT.

**Restrictions:** CoreSlicer is not licensed for clinical or commercial use.

## Additional files

Additional file 1: Algorithm for computer-assisted adipose and muscle tissue segmentation on abdominal CT scan images. Describes the elemental segmentation algorithms used to showcase CoreSlicer’s functionality on www.coreslicer.com. (DOCX 100 kb)

Additional file 2: **Figure S1.** Illustration of muscle and adipose tissue boundary detection in pseudo-polar coordinates. (PDF 1579 kb)

Additional file 3: **Figure S2.** Bland-Altman plot of difference in VFA, SFA and TLMA for manual measurements in CoreSlicer by Observers A versus automated segmentation. (PDF 836 kb)

## Abbreviations

BMI: Body mass index
CT: Computed tomography
DICOM: Digital Imaging and Communications in Medicine
GPU: Graphics processing unit
MIA: Medical image analysis
MR: Magnetic resonance
PMA: Psoas muscle area
ROI: Region of interest
SFA: Subcutaneous adipose tissue area
TLMA: Total skeletal muscle area
VFA: Visceral adipose tissue area
