# Supplementary material for: CoreSlicer: a web toolkit for analytic morphomics
Source: BMC Med Imaging. 2019 Feb 11;19:15. doi: 10.1186/s12880-019-0316-6 (PMC6371488; doi:10.1186/s12880-019-0316-6)
Supplement: Supplementary file 1 — Algorithm for computer-assisted adipose and muscle tissue segmentation on abdominal CT scan images. Describes the elemental segmentation algorithms used to showcase CoreSlicer’s functionality on www.coreslicer.com. (DOCX 100 kb) [file 12880_2019_316_MOESM1_ESM.docx]

**Supplemental Material. Algorithm for computer-assisted adipose and muscle tissue segmentation on abdominal CT scan images.**

For computer-assisted segmentation of VFA, SFA and TLMA, an automated algorithm was developed to identify boundaries between muscle and adipose tissue compartments on axial abdominal CT images (Supplemental Figure 1).

*1. Segmentation Boundaries.* An iterative edge fitting procedure was used to track segmentation boundaries between muscle and adipose tissue compartments on abdominal CT images. The boundaries of interest were: 1) along the outer margin of the abdominal wall and back muscles; 2) along the inner margin of the abdominal wall and back muscles; and 3) along the smallest region containing the entire visceral fat. VFA, SFA and TLMA were determined by combining the cross-sectional areas delimited by the resulting boundaries.

*2. Normalization and Alignment.* Prior to segmentation, single-slice CT scan images in DICOM format were cropped, resized to 512x512 pixels, and aligned with a reference image. Image alignment was performed via an affine transform with maximization of the enhanced correlation coefficient using the OpenCV software package (version 3.0). The maximal number of iterations was set at 500. The reference alignment image was excluded from further use during testing and training.

*3. Image Projection.* Following alignment, images were projected using a pseudo-polar transform similar to the rubber sheet transform used in the iris recognition literature. Each point in the Cartesian representation of the CT image was assigned dimensionless coordinates $(r,\theta)$ with respect to a central reference point, defined in this study as the most anterior point of the L4 vertebral body. The vertebral body was automatically segmented via a thresholding and binary filling operation. The projection is mathematically described as follows:

$$I(x\left( r,\theta\right), y\left( r,\theta\right))\to I\left( r,\theta\right)$$

$$x\left( r,\theta\right)=rx_{b}\left( \theta\right)$$

$$y\left( r,\theta\right)=ry_{b}\left( \theta\right)$$

where $(x, y)$ are the Cartesian coordinates, $(r,\theta)$ are the polar coordinates, and $(x_{b}(\theta), y_{b}(\theta))$ are the coordinates of the boundary points in the $\theta$ direction [35].

*4. Boundary Fitting Procedure.* Let there be a collection of *N* normalized, aligned and transformed training images, where each image *n* is represented by an intensity matrix $\mathbf{I}_{n}$; a set of *K* boundaries of interest for each image, where boundary *k* of image *n* is represented by the vector $\mathbf{e}_{k}^{n}$; a set of fitting parameters for each boundary *k* represented as $\mathbf{p}_{k}$; a family of fitting functions $e_{k}$ such that $\mathbf{e}_{k}^{n}$ $=e_{k}(\mathbf{I}_{n},\mathbf{p}_{k})$; a family of reverse-projection functions $a_{k}$ that transform boundaries into their corresponding segmented regions $\mathbf{A}_{k}^{n}$, such that $\mathbf{A}_{k}^{n}=a_{k}(\mathbf{e}_{k}^{n})$; and a set of ground truth segmentation areas $\mathbf{R}_{k}^{n}$. The boundary fitting problem is to find the parameters $\mathbf{p}_{k}$ that minimize the segmentation error over all images in the training set:

$$\arg\min_{\mathbf{p}_{\boldsymbol{k}}}\sum_{n=1}^{N} J_{\delta}(\mathbf{A}_{k}^{n},\mathbf{R}_{k}^{n})$$

where $J_{\delta}(A,B)$ is the inverse Jaccard coefficient or Jaccard distance, defined as:

$$J_{\delta}(A,B)=1-\frac{\left| A\cup B \right|-\left| A\cap B \right|}{|A\cup B|}$$

*5. Boundary Fitting Function.* The boundary fitting function $e_{k}(\mathbf{I})$ is obtained by composition of a denoising function $f_{k}\left( \mathbf{I,p} \right)$, detection function $g_{k}\left( \mathbf{I}_{d}\mathbf{,e',p} \right)$**,** and smoothing function $h\left( \mathbf{d,p} \right)$:

$$\mathbf{e}_{n}^{k}\mathbf{=}e_{k}\left( \mathbf{I}_{n} \right)\mathbf{=}h\left( g\left( f\left( \mathbf{I,}\mathbf{p}_{\mathbf{k}} \right),\mathbf{e}_{\boldsymbol{n}}^{\mathbf{k-1}}\mathbf{,}\mathbf{p}_{\mathbf{k}} \right)\mathbf{,}\mathbf{p}_{\mathbf{k}} \right)$$

where$\mathbf{I}$ is the intensity image, $\mathbf{p}$ is the set of edge detection parameters, **d** is the set of unsmoothed edge points, $\mathbf{e}_{n}^{k}$ is the smoothed edge *k* as detected in image *n*, and $\mathbf{e'}$**=**$\mathbf{e}_{n}^{k-1}$ is the edge detected during the previous iteration of the algorithm.

*6. Boundary Fitting Operators.* Various combinations of denoising filters, edge detectors, and smoothing filters were evaluated in preliminary experiments. In the final model, a median filter was used for denoising, a threshold filter was used for edge detection, and a percentile filter was used for edge smoothing.

$$f\left( \mathbf{I,p} \right)=\sum_{(i,j)} \mathrm{median}\left\{ \mathbf{I}\left[ i,j \right], \left( i,j \right)\epsilon N_{i}^{p_{1}} \right\}$$

$$g\left( \mathbf{I}_{d}\mathbf{,}\mathbf{e}_{k-1}\mathbf{,p} \right)\boldsymbol{=}\sum_{i} \arg\min_{\mathbf{p}_{\mathbf{2}}}\left\{ p_{1}<\mathbf{I}_{d}\left[ i,\mathbf{e}_{n-1}\left[ i \right]+p_{2} \right]<p_{4} \right\}$$

$$h\left( \mathbf{d,p} \right)=\sum_{i} \mathrm{percentile}\left\{ \mathbf{T}\left[ i,j \right], p_{5}\left( i,j \right)\epsilon N_{i}^{p_{6}} \right\}$$

where $p_{1}>0$ is the neighborhood size for the 2D median filter,

$p_{2}>0$, $p_{3}$, $p_{4}$ are the offset and thresholds for the edge detector,

$0<p_{5}<100$ and $p_{6}>0$ are the percentile and neighborhood size for the percentile filter.

The median filter was found to be a good choice for denoising of its ability to reduce noise while preserving edges for subsequent detection steps. The threshold edge detector was found to outperform gradient methods, in large part because these methods fail to capture edge intensity information, which is important in delineating tissue boundaries in CT images. Finally, the percentile filter was found to be a good choice for curve smoothing because it is robust to outliers, and allows for flexible tuning by varying the percentile parameter.

*7. Parameter Estimation.* The parameters $\mathbf{p}_{k}$ were learned sequentially for each boundary *k* using simulated annealing over the training set, which consisted of 10 CT images separate from the images used for testing. Simulated annealing was performed with SciPy (version 0.17) using the “fast” annealing schedule, with default values for other parameters.

**Validation**

LM performed measurements of VFA, SFA and TLMA once using the manual drawing tools in the CoreSlicer web interface, and once using a CoreSlicer plugin implementing the proposed algorithm (version 1.0). DICOM images were imported into the CoreSlicer web interface. The axial image corresponding to the top of the L4 level was identified using a mid-sagittal reconstruction view. For manual measurements, the threshold brush tool was used to trace the cross-sectional areas of interest after selecting the corresponding Hounsfield unit range. For automated measurements, the full-slice segmentation tool was used to generate the cross-sectional areas of interest; manual corrections were not applied. Result sets were exported in CSV format using the “Save” function.

Results are summarized in the table below. Mean absolute differences in cross-sectional areas were -5.1 cm^2^ (95% CI -7.5 to -2.7 cm^2^) for VFA, -7.1 cm^2^ (95% CI -9.2 to -4.8 cm^2^) for SFA, and -1.2 cm^2^ (95% CI -3.3 to 0.8 cm^2^) for TLMA. Mean Jaccard coefficients for automated vs. manual segmentation were 0.88 (95% CI 0.87 to 0.89) for VFA, 0.92 (95% CI 0.91 to 0.93) for SFA and 0.88 (95% CI 0.87 to 0.89) for TLMA (Supplemental Figure 2).

**Comparison of Automated Measurements of VFA, SFA and TLMA
Versus Manual Results in CoreSlicer**

| **Measurement** | **Mean difference**  **(95% CI)** | **Jaccard coefficient  (95% CI)** |
| --- | --- | --- |
| VFA | -7.1 cm^2^ (-9.2 to -4.8 cm^2^) | 0.88 (0.87 to 0.89) |
| SFA | -1.2 cm^2^ (-3.3 to 0.8 cm^2^) | 0.92 (0.91 to 0.93) |
| TLMA | -5.1 cm^2^ (-7.5 to -2.7 cm^2^) | 0.88 (0.87 to 0.89) |
