# Supplementary figures and images for: CoreSlicer: a web toolkit for analytic morphomics
Source: BMC Med Imaging. 2019 Feb 11;19:15. doi: 10.1186/s12880-019-0316-6 (PMC6371488; doi:10.1186/s12880-019-0316-6)

a

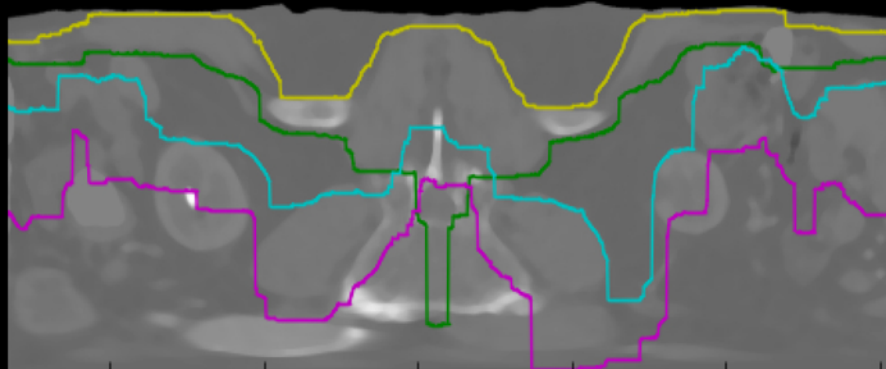

b

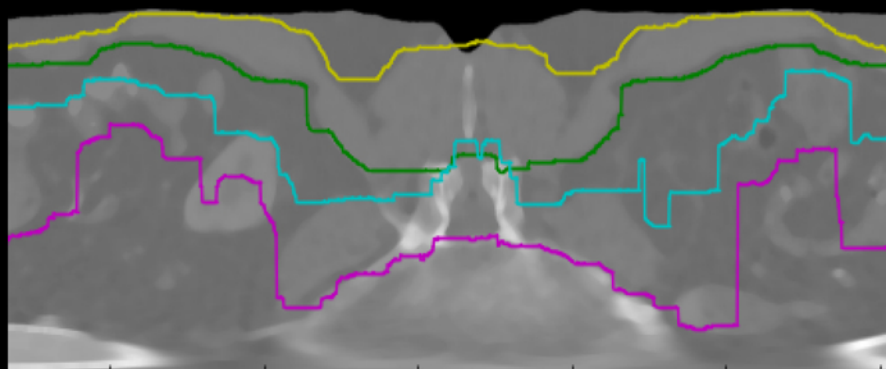

c

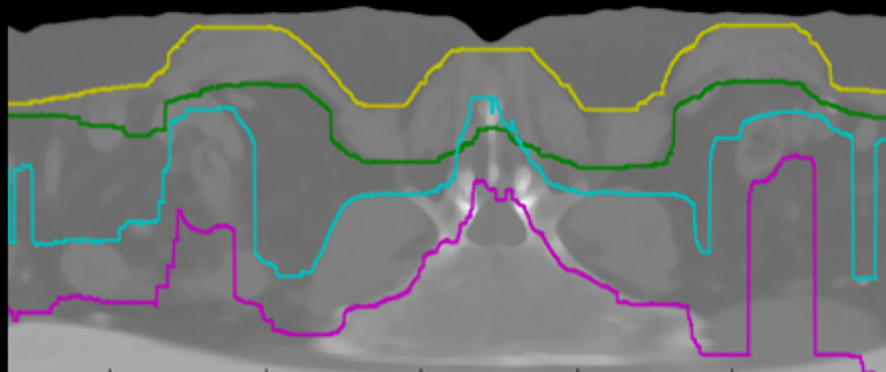

Supplement: Supplementary file 2 — Figure S1. Illustration of muscle and adipose tissue boundary detection in pseudo-polar coordinates. (PDF 1579 kb) [file 12880_2019_316_MOESM2_ESM.pdf]
