# Supplementary material for: CoreSlicer: a web toolkit for analytic morphomics
Source: BMC Med Imaging. 2019 Feb 11;19:15. doi: 10.1186/s12880-019-0316-6 (PMC6371488; doi:10.1186/s12880-019-0316-6)
Supplement: Supplementary file 3 — Figure S2. Bland-Altman plot of difference in VFA, SFA and TLMA for manual measurements in CoreSlicer by Observers A versus automated segmentation. (PDF 836 kb) [file 12880_2019_316_MOESM3_ESM.pdf]

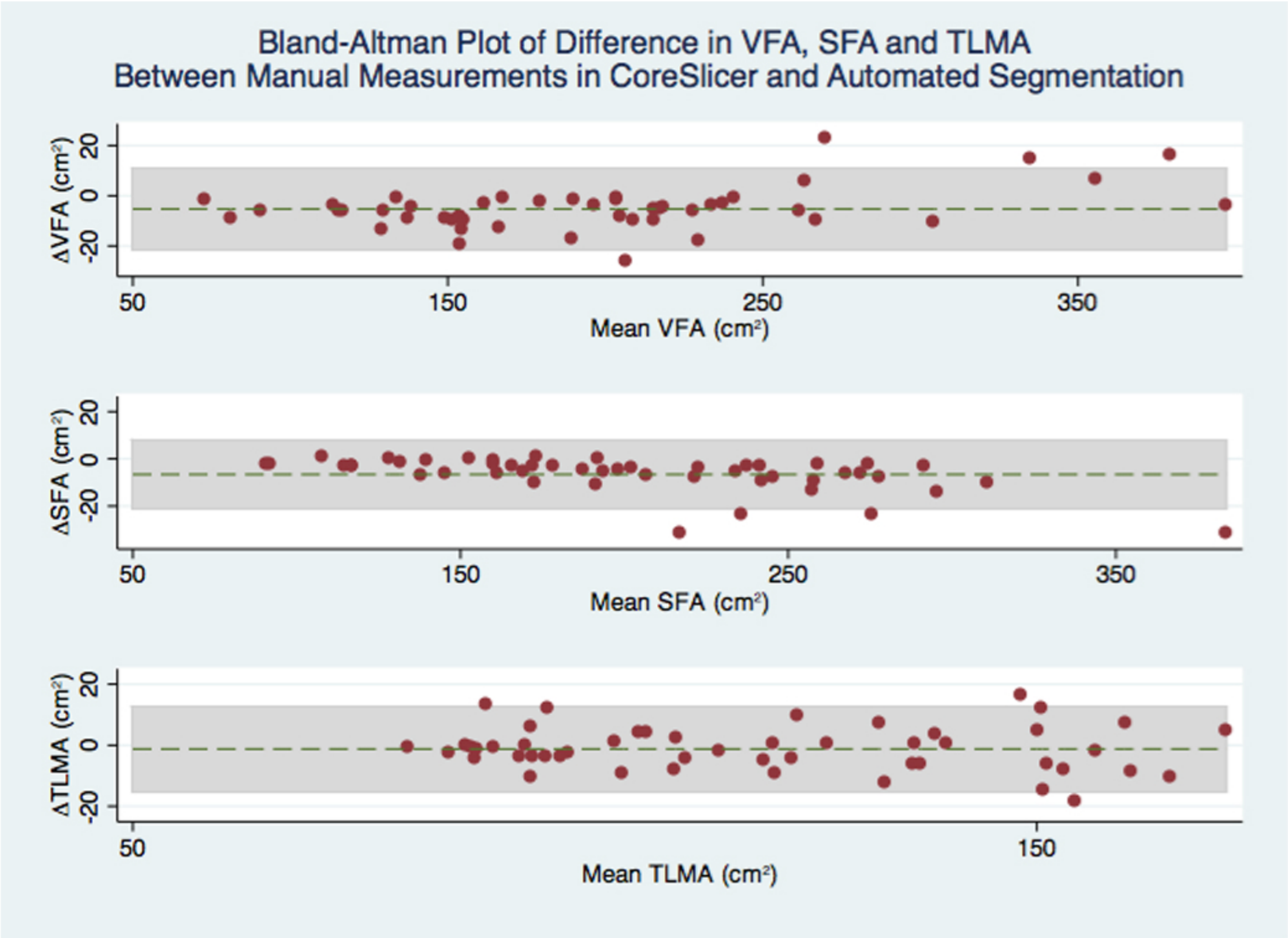
Bland-Altman Plot of Difference in VFA, SFA and TLMA
Between Manual Measurements in CoreSlicer and Automated Segmentation
ΔVFA (cm²)
20
0
-20
50
150
250
350
Mean VFA (cm²)
ΔSFA (cm²)
20
0
-20
50
150
250
350
Mean SFA (cm²)
ΔTLMA (cm²)
20
0
-20
50
150
Mean TLMA (cm²)
